# Deconvoluting the diversity of within-host pathogen strains in a multi-locus sequence typing framework

**DOI:** 10.1186/s12859-019-3204-8

**Published:** 2019-12-17

**Authors:** Guo Liang Gan, Elijah Willie, Cedric Chauve, Leonid Chindelevitch

**Affiliations:** 1 School of Computing Science, Simon Fraser University, 8888 University Drive, Burnaby (BC), V5A 1S6 Canada; 2 Department of Mathematics, Simon Fraser University, 8888 University Drive, Burnaby (BC), V5A 1S6 Canada; 3 LaBRI, Université de Bordeaux, 351 Cours de la Libération, Talence, 33405 France

**Keywords:** Multi-Locus Sequence Typing, Bacterial diversity, Integer Linear Programming

## Abstract

**Background:**

Bacterial pathogens exhibit an impressive amount of genomic diversity. This diversity can be informative of evolutionary adaptations, host-pathogen interactions, and disease transmission patterns. However, capturing this diversity directly from biological samples is challenging.

**Results:**

We introduce a framework for understanding the within-host diversity of a pathogen using multi-locus sequence types (MLST) from whole-genome sequencing (WGS) data. Our approach consists of two stages. First we process each sample individually by assigning it, for each locus in the MLST scheme, a set of alleles and a proportion for each allele. Next, we associate to each sample a set of strain types using the alleles and the strain proportions obtained in the first step. We achieve this by using the smallest possible number of previously unobserved strains across all samples, while using those unobserved strains which are as close to the observed ones as possible, at the same time respecting the allele proportions as closely as possible. We solve both problems using mixed integer linear programming (MILP). Our method performs accurately on simulated data and generates results on a real data set of *Borrelia burgdorferi* genomes suggesting a high level of diversity for this pathogen.

**Conclusions:**

Our approach can apply to any bacterial pathogen with an MLST scheme, even though we developed it with *Borrelia burgdorferi*, the etiological agent of Lyme disease, in mind. Our work paves the way for robust strain typing in the presence of within-host heterogeneity, overcoming an essential challenge currently not addressed by any existing methodology for pathogen genomics.

## Background

The study of bacterial pathogens has revealed an impressive genetic diversity that had not been fully suspected prior to the advent of genome sequencing technologies. This diversity may indicate an adaptive response to challenges such as the variability in host genetics, environmental conditions, and, in the case of pathogens affecting humans, the introduction of antibacterial drugs [1–4].

One bacterial pathogen that is particularly well-known for its genetic diversity is *Borrelia burgdorferi*, the etiological agent of Lyme disease. It has been found that up to six genetically different strains can affect a single host [5, 6]. Furthermore, this diversity may result from both clonal evolution within the host as well as multiple infection events [7]. Unfortunately, techniques such as bacterial culture are difficult to apply to reveal the whole range of diversity in bacteria like *B. burgdorferi*, a situation common to many bacterial pathogens. Next-generation sequencing (NGS) techniques such as whole-genome sequencing (WGS) with short reads have revolutionized our ability to investigate the genomic diversity of bacteria and other organisms [8]. Recently, an adaptation of WGS technology to *B. burgdorferi*, called whole-genome capture, has been proposed which is able to reliably filter out irrelevant DNA (such as host DNA) [9]. This novel approach for generating sequence data for *B. burgdorferi* nicely complements a highly reproducible strain-typing scheme known as multi-locus sequence typing (MLST), which has been developed and found to be useful for different pathogens in a number of contexts [10]. MLST is a summary of the bacterial genotype using the alleles of several (typically 6 to 9) housekeeping genes, which may be further grouped into closely related strain types. In the case of *B. burgdorferi*, several hundred strain types have been characterized using the MLST scheme developed in [11], while only 111 fully sequenced *B. burgdorferi* genomes^1^ are currently available in the NCBI databases. MLST strain types thus provide a finer-grained picture of the strain diversity of this pathogen, which motivates the need for developing novel diversity estimation methods that combine NGS data and the wealth of strain types already characterized by MLST.

In principle, this problem is a special instance of estimating the diversity and abundance of microbial strains from metagenomics data, a problem for which several accurate methods have recently been developed (e.g. [12–14]). De novo methods, such as DESMAN [12], cannot take advantage of known reference strains or alleles and are likely to be confounded by the high similarity observed between strain types. Other methods such as strainEST [13] are able to consider a large set of reference genomes, which in our case can be defined by the concatenated allele sequences of the known *B. burgdorferi* strain types, but again, their diversity models are not well adapted to handle the very high similarity between strain types. Moreover, none of the reference-based methods consider the detection of novel strain types.

We introduce the first paradigm for extracting MLST information in the presence of within-host heterogeneity, which is also able to simultaneously take multiple samples into account and detect novel strains. Our method is based on mixed integer linear programming (MILP), and consists of two main stages. It starts by filtering the short reads in each sample, selecting those that closely match known alleles in at least one of the housekeeping genes in the MLST scheme, and then assigns fractional abundances to each allele of each gene, ensuring that as few such alleles as possible are used to explain the data. In the second stage, it assigns combinations of these alleles, with corresponding proportions, to each sample, while maximizing the usage of known strains and minimizing the number of novel strains, a parsimony-based approach that has been shown to perform well in related contexts [15].

We evaluate our approach on simulated samples and find that it is accurate in identifying both the fractional allele composition at each housekeeping gene, as well as the complete strain types present in each sample. We then apply it to a dataset of 24 real tick samples containing *B. burgdorferi* extracted via whole-genome capture, and find a substantial amount of diversity, as well as a number of new strains. In conclusion, our work provides a robust and reproducible pipeline for accurate strain typing via MLST from WGS data even in the presence of substantial within-host heterogeneity.

## Methods

**Fig. 1:**
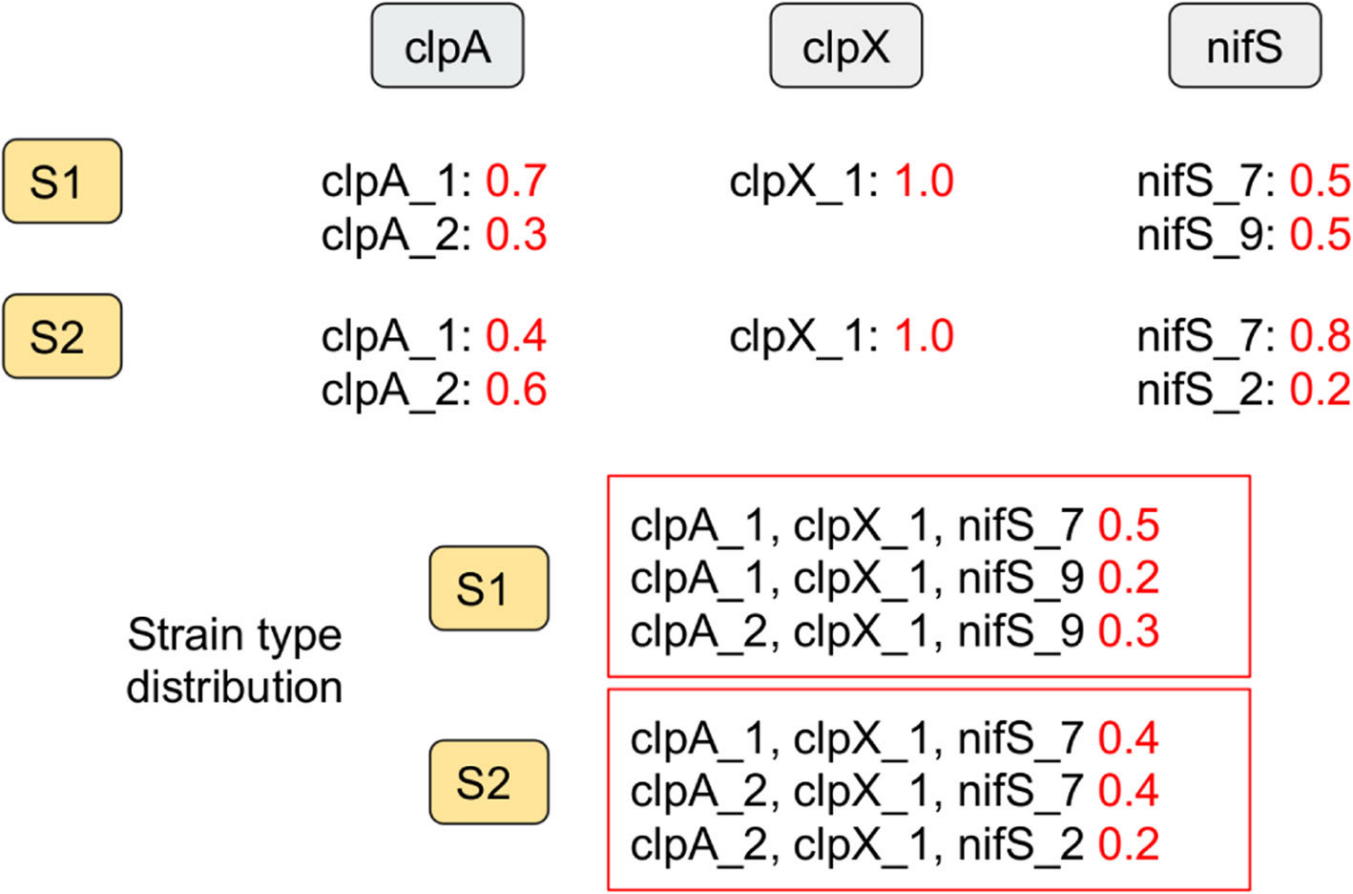
A dataset with two samples and an MLST scheme of three loci (genes clpA, clpX, nifS). The strain type distributions require 5 different strains as the strain (clpA_1,clpX_1, nifS_7) appears in both distributions

**Terminology.**An *MLST scheme* is composed of a set of loci together with a database of known alleles for each locus [16]. An *allele distribution* for a given locus is a set of alleles for this locus together with a proportion assigned to each allele; the proportions must be non-negative and add up to 1. A *strain type* is an assignment of a specific allele to each gene of the MLST scheme. A *strain type distribution* is a set of strain types together with a proportion assigned to each strain type; the proportions must once again be non-negative and add up to 1. A *sample* is a WGS dataset obtained from a single host that contains the sequence data from one or several pathogen strains present in the host (see Fig. 1).

**Data.**In the present work we use the traditional *B. burgdorferi* MLST scheme [11] composed of 8 housekeeping genes having a combined total of 1726 known alleles. For each locus, the various known alleles differ from one another primarily by single nucleotide polymorphisms (SNPs), with small indels also appearing in 4 out of the 8 genes. The number of known strain types is 753.

**Problems and contribution overview.**The problems we address in this work take as input (1) an MLST scheme together with databases of known alleles and strain types and (2) WGS data for a set of samples that are mapped using a short-read mapper of choice onto the database of known alleles for the provided MLST scheme. It then proceeds in two stages, each addressing a specific problems:

- The Allele Diversity Problem. For a given sample and a given locus of the MLST scheme, given the mappings of DNA reads onto the known alleles for this locus, detect the alleles present in the sample and the corresponding allele distribution.
- The Strain Diversity Problem. Given a set of samples and an allele distribution for each locus at each sample, compute a strain type distribution per sample that requires the smallest number of novel strain types *among all considered samples*, which are as similar as possible to known strains.

### The Allele Diversity Problem

We formulate the problem of allele detection as a variant of the Set Cover problem as follows. The input of the Allele Diversity Problem (ADP) is composed of a set of *m* reads $\mathcal {R}= \{r_{1},\dots \,r_{m}\}$, a set of *n* alleles $\mathcal {A} = \{a_{1},\dots,a_{n}\}$ for the chosen locus, and a set of mappings of the reads onto the alleles, encoded by a matrix *M*, where *m*_*ij*_ is the sum of the normalized Phred scores of the mismatched bases in the mapping of read *r*_*i*_ onto allele *a*_*j*_ (we set it to *∞* if *r*_*i*_ does not map onto *a*_*j*_). For instance, assuming that the range of acceptable Phred scores is from 33 to 126, if read *r*_*i*_ maps to allele *a*_*j*_ with 2 mismatches with base quality scores of 60 and 80, respectively, then $m_{ij}=\frac {60-33}{126-33} + \frac {80-33}{126-33} = 0.796$. Each allele *a*_*j*_ implicitly defines a subset of $\mathcal {R}$ (the reads aligning with the allele), with each read *r*_*i*_ being weighted by *m*_*ij*_. Informally, we then aim at selecting a subset of alleles covering the set of reads, while minimizing the sum of the number of required alleles and the sum of the corresponding weights. The ADP is thus very similar to the Uncapacitated Facility Location Problem, and we discuss this observation in Additional file 1.

Formally, we define an edge-weighted bipartite graph whose vertex set is $\mathcal {R} \cup \mathcal {A}$ and whose weighted incidence matrix is *M*. A *read cover* is a subset of edges of this graph such that each read belongs to exactly one edge; the cost of a read cover is the number of allele vertices it is incident to plus the sum of the weights of the edges in the cover. The ADP aims at finding a read cover of minimum weight, the allele vertices incident on the edges of the cover representing the selected alleles.

#### **Theorem 1**

The Allele Diversity Problem is NP-hard.

The proof of Theorem 1 relies on a reduction from the 3-dimensional matching problem and is provided in Additional file 1. Before describing our ILP we comment on the relevance of our formulation for selecting a set of alleles from short reads. Our objective function aims to minimize the sum of the number of alleles and the weight of each read based on the Phred scores; the latter part aims at explaining the data (reads) using as few errors/mismatches as possible, accounting for the base quality score of the mismatches, while the former part ensures that an allele is not introduced unnecessarily to reduce the contribution of the mismatches and their quality for a small number of reads. Our experiments on simulated data show that this objective function leads to extremely accurate results.

**An Integer Linear Program for the Allele Diversity Problem.**First we introduce the following notation: *R*_*j*_={*r*_*i*_:*m*_*ij*_≠*∞*} represents the set of reads mapping onto allele *a*_*j*_ (i.e. covered by allele *a*_*j*_), and $\ M_{i} = \{m_{ij} | 1 \leq j \leq n\} - \{\infty \} = \{q_{i1},..., q_{i|M_{i}|}\}$ represents the distinct summed Phred scores for read *r*_*i*_. The decision variables of the ILP are:

- *x*_*j*_=1 if allele *a*_*j*_ is chosen, and 0 otherwise.
- *y*_*ik*_=1 if a mapping of read *r*_*i*_ with score *q*_*ik*_ is chosen, and 0 otherwise.

The objective function is $\min \!\left (\! \sum _{i=1}^{|\mathcal {R}|}\! \sum _{k=1}^{|M_{i}|} q_{ik}\! \cdot \! y_{ik}\! +\!\! \sum _{j=1}^{n}\! x_{j}\!\right)$.

Finally, the constraints of the ILP are the following ones:

- If *y*_*ik*_=1, there exists some allele *a*_*j*_ onto which *r*_*i*_ maps with score *q*_*ik*_.
- There is a unique score with which read *r*_*i*_ is mapped onto the selected alleles.

These constraints can be represented as follows:

$$\sum_{\{j\ |\ r_{i} \in R_{j}, m_{ij} = q_{ik}\}} x_{j} \geq y_{ik} \, \forall \, i,k \hspace{1cm}\sum_{k=1}^{|M_{i}|} y_{ik} = 1 \, \forall \, i. $$

**Post-processing.**If the above 0-1 ILP has multiple optimal solutions, we resort to a likelihood based method to select one, namely GAML [17], a probabilistic model for genome assembly. Given a set of solutions where each solution represents a set of alleles, we measure the likelihood of observing the set of reads given a solution and pick the solution which maximizes the likelihood criterion. If there are multiple solutions maximizing the likelihood criterion, we pick one arbitrarily.

**Computing allele proportions.**Finally, once the alleles have been identified for a given locus, we compute the proportion of each allele. The principle is to assign a weight to each allele based on the read mappings (edges) selected by the ILP, and to normalize these weights to obtain proportions. First, we filter out any read that maps equally well (i.e. with the same score *k*) onto all selected alleles. Then every chosen allele gets an initial weight of 0. Next, for every non-discarded read, say *r*_*i*_, we consider all the alleles it maps onto with optimal score (say *q*_*ik*_ if *y*_*ik*_=1); assuming there are *h* such alleles, we increase the weight of each by 1/*h*. We then normalize the weights of the alleles to define their respective proportions.

### The Strain Diversity Problem

Once the alleles present in each sample and their proportions have been identified, this information is passed to the second stage of the pipeline. Its goal is to compute strain types and proportions in all samples *jointly*, minimizing the number of novel strains required to explain the given allele distributions plus an error term measuring the total discrepancy between each given allele proportion and the proportions of strains having this allele. The rationale behind minimizing the number of new strains is driven by parsimony considerations; we would like to explain the data present in all samples using known strains as much as possible. The error terms allow some flexibility to modify the allele proportions by bounding each error to be ≤*ε* (in our analysis we set the bound to *ε*=0.1, or 10%).

**The Strain Diversity Problem: problem definition and tractability.**The Strain Diversity Problem (SDP) can be defined as follows. It takes as input four elements: (1) the set *G*_*ij*_={*g*_*ij*1_,*g*_*ij*2_,… } of all alleles selected for locus *j* in sample *i* (2) the set *P*_*ij*_={*p*_*ij*1_,*p*_*ij*2_,… } of proportions of these alleles, (3) a database *Ω* of known strain types, (4) an error bound *ε*∈[0,1]. From now on, we assume that there are *ℓ* loci and *m* samples.

From this input, we generate the set of all possible strain types for each sample *i*, defined as the Cartesian product *G*_*i*1_×*G*_*i*2_×⋯×*G*_*iℓ*_ which we denote by $V_{i} = \{V_{i1}, V_{i2}, \dots,V_{iH_{i}}\}$ with $H_{i} = \prod _{j=1}^{\ell }|G_{ij}|$. We also denote by *K* the number of strain types that appear in at least one *V*_*i*_ and we define the set $\mathcal {S}=\{S_{1}, \dots, S_{K}\}$ of all such strain types. We assign a weight *w*_*j*_ to each $\mathcal {S}_{j} \in \mathcal {S}$, where $w_{j} = N \cdot \min _{\{s \in \Omega \}} d(s, \mathcal {S}_{j})$, where *d* is the edit distance metric and *N* is a normalization constant that rescales the weights to the interval [0,1]. These weights measure the distance to the closest known strain; the strains in *Ω* are assigned a weight of 0.

A solution to the SDP is fully described by assigning to every strain type *V*_*ih*_ from *V*_*i*_ a proportion *π*_*ih*_ for this strain type in sample *i* (where *π*_*ih*_ is 0 if the strain type is deemed to be absent from sample *i*). A strain type from $\mathcal {S} \setminus \Omega $ is said to be present in a solution if it is given a non-zero proportion in at least one sample; we denote by $\mathcal {S}_{n}$ the set of such novel strain types. The cost of a solution is then defined as

1 $$  \sum_{\{h | \mathcal{S}_{h} \in \mathcal{S}_{n} \}} w_{h} + \sum_{i,j} e_{ij}  $$

where the latter term of the cost represents the deviation from the input alleles proportions for sample *i* at locus *j*. This cost function penalizes the introduction of novel strains that are very different from known strains and the error introduced in the proportions of the selected alleles. The SDP aims at finding a solution of minimum cost, i.e. one that explains the provided allele distributions as much as possible with known strains and novel strains which are close to the known strains, and also adheres to the desired proportions as closely as possible. As expected, this problem is intractable; its decision version is proven to be NP-complete in Additional file 1, by a reduction from the 3-partition problem.

#### **Theorem 2**

The Strain Diversity Problem is NP-hard.

**An MILP for the Strain Diversity Problem.**We now describe an MILP that solves the SDP. The decision variables of the MILP are the following:

- Binary variables *a*_*k*_,1≤*k*≤*K*, where *a*_*k*_=1 if strain type *S*_*k*_ is chosen to explain the observed allele distribution in at least one sample, and 0 otherwise.
- Proportion variables *π*_*ih*_ encoding the proportion of strain type *V*_*ih*_ in sample *i*; their values are constrained to be in [0,1].
- Variables *e*_*ijk*_∈[0,*ε*] encoding the absolute error of the observed proportion *p*_*ijk*_ of allele *g*_*ijk*_ for locus *j* in sample *i* from the assigned proportions, in sample *i*, of the strain types containing this allele.

The objective function of the MILP is

2 $$ \min\left(\sum_{\{k\ |\ S_{k} \notin \Omega\}} w_{k} a_{k} + \sum_{i,j,k} e_{ijk}\right)  $$

Finally the constraints of the MILP are the following:

- For any allele *g*_*ijk*_∈*G*_*ij*_, the sum of the proportions of the strain types from *V*_*i*_ that contain this allele, denoted *ν*_*ijk*_, belongs to [*p*_*ijk*_−*ε*,*p*_*ijk*_+*ε*].
- For each sample *i*, the strain type proportions must form a distribution: $\sum _{h=1}^{H_{i}}\pi _{ih} = 1$.
- If the assigned proportion for some strain type *V*_*ih*_=*S*_*k*_ in a sample *i* is non-zero, then *S*_*k*_ must be chosen: *a*_*k*_≥*π*_*ih*_.
- Conversely, if a strain is chosen, it must be assigned a non-zero proportion:
  $$0 \leq a_{k} - \frac{1}{|\{\pi_{ih}\ |\ V_{ih} = S_{k}\}|} \cdot \sum_{\{ (i,h) | V_{ih} = S_{k}\}} \pi_{ih} \leq 1 - \delta$$
  where *δ* is a tolerance chosen to match the smallest allowed proportion; we use *δ*=0.001. This constraint is needed because the binary decision variables for the usage of existing strains have coefficient 0 in the objective function, so setting these variables to 1 will not incur any cost in the objective function. If we do not impose such a constraint, we could end up with an incorrect solution where some existing strains have zero proportions, while the strain usage variables are set to 1, which would then need to be post-processed. Including this constraint eliminates the possibility of such a spurious solution.
- The absolute error between the input proportion and the assigned proportion for allele *g*_*ijk*_ for locus *j* in sample *i*: *e*_*ijk*_=|*p*_*ijk*_−*ν*_*ijk*_|. This is encoded by the following 2 constraints: *e*_*ijk*_≥*T*_*ijk*_−*p*_*ijk*_ and *e*_*ijk*_≥*p*_*ijk*_−*T*_*ijk*_ where $T_{ijk}=\sum _{\{k\ |\ g_{ijk} \in V_{ik}\}}\pi _{ik}$. Note that since *e*_*ijk*_ is part of the objective function to be minimized, it will be equal to the error in any optimal solution.

### Implementation

All scripts are written in Python 2.7. Both ILPs are formulated and solved using the Python API of IBM’s CPLEX 12.6.3.0. For the ADP, each sample and each locus may require a different number of variables in the ILP. To evaluate the practical resources requirements of our ILP, we choose the sample SRR2034336, which has the largest number of reads among our samples. The average number of variables across each gene for this sample is 20,112, the maximum RAM usage is ∼1.5GB, and the time taken for all 8 genes is ∼33 min on a 4 CPUs Intel^®^ Xeon^®^ machine. The total time taken for each sample is presented in Additional file 1. For the MILP solving the SDP on all 30 samples, there are a total of 21,885 variables, with 10,682 strain type variables, 10,795 proportion variables and 408 error variables. Due to the computational complexity of the MILP, we output a solution as long as the relative gap tolerance is within 10% and after a time limit of 24 h. Our code is publicly available at https://github.com/WGS-TB/MLST.

### Data simulation

Given the absence of benchmarks available for estimating diversity at the level of precision considered in this work, we conducted several simulations. All reads are simulated using ART [18], following the characteristics of the reads from the real data set described in “Application to real data” section.

**ADP simulation.**For each locus of the *Borrelia* MLST scheme, we drew a random number *k*∈[2,7], selected a random allele from the database and selected *k*−1 other alleles, each at edit distance at most *d* (a given parameter) from the first one chosen. Next, we randomly assigned proportions to each selected allele, which sum up to 1, then generated reads with coverage *c*. To align the simulated reads to the alleles of the database, we used Bowtie v0.12.7 [19]. We used parameters *c*∈{30,100,300} and *d*∈{5,10,15,20,25} and we ran 40 simulations for each combination of these parameters. For this experiment, we compared our results with the results obtained with Kallisto [20], a recent method for isoform abundance estimation that has also been applied to metagenomics.

**SDP simulation**For this simulation we selected random strain type distributions and tested the ability of our SDP method to recover the true diversity given perfect allele calls. We considered 5 different mechanisms to generate strain types distributions. EvoMod1: We select a random existing strain *S*, which is then mutated *m*=2 times to obtain a new strain *S*^′^, where each mutation results in an allele which has edit distance at most *d*=15 from the original allele in *S*. The total number of strains simulated is 2 (1 existing and 1 novel). EvoMod2: We repeat EvoMod1 in parallel from two starting existing strains. The total number of strains simulated is 4 (2 existing and 2 novel). EvoMod2e/EvoMod2n: We apply EvoMod2 then remove a random existing/novel strain. EvoMod3: we apply EvoMod2, then apply a recombination (allele exchange) event on two randomly chosen strains out of the 4 available strains. For all experiments, we assigned random proportions to the chosen strains.

**Full pipeline simulation.**We generated strain type distributions as in the SDP simulations above, then generated reads as in the ADP simulations. The generated reads were then fed to the ADP solver, and the ADP results were provided as input to the SDP solver. We compared our pipeline with strainEST [13], a recent method to estimate the strain composition and abundance in metagenomics datasets. However, strainEST does not predict novel strain types. Hence, to complement EvoMod1, 2, 2e and 2n, we added an additional simulation where we randomly pick *k*={1,2} existing strains and assign them random proportions.

**Statistics.**For each experiment, we recorded the following statistics: Precision, Recall and Total Variation Distance. Precision and recall are defined as $\frac {TP}{TP+FP}$ and $\frac {TP}{TP+FN}$, where *TP*, *FP*, *FN* are the number of true positive calls, false positive calls, and false negative calls, respectively. The Total Variation Distance (TVD) [21, p. 50] is defined as $TVD = \frac {1}{2}\sum _{a \in S}|Pred(a) - True(a)|$, where *Pred* and *True* are the predicted distribution and the true distribution, respectively, and *S* is the set of all possible outcomes. The TVD basically describes the average amount of distribution to “move” from *Pred* to *True* or vice versa.

The statistics described above rely on a stringent measure of accuracy in calling alleles, strain types or proportions. For example, a novel strain type called which differs from the true simulated strain type by a single SNP would be considered as a False Positive. To account for this, we considered 3 additional statistics: Earth-Mover’s distance (EMD), soft-precision and soft-recall. Soft precision and soft recall are similar to precision and recall, however, a strain is considered a TP if it differs from the true strain type by at most 5 SNPs. The EMD [22] is similar in principle to the TVD, but is more refined as it considers the edit distances between strains and is commonly used in genomics to evaluate haplotype reconstruction methods [23]. We provide a full definition in Additional file 1.

## Results

### Simulated data

We describe several sets of experiments based on simulated data. In the first one we evaluate our method for the ADP problem and compare it with Kallisto. In the second experiment, we evaluate our method for the SDP, using simulated allele frequencies, i.e. perfect input to the SDP, and 4 different evolutionary models explaining the diversity within a sample, from a simple model based on within-host mutations to a complex model based on co-infection and recombination. We then repeat the same experiment using simulated short reads, to evaluate our pipeline on ADP + SDP. Finally, we compare our method to strainEST using simulated datasets with no novel strains (the ideal case for strainEST) and then datasets simulated using evolutionary modes identical to the ones in the previous experiment.

**ADP simulation.**Table 1 shows the performance of our method. Overall, our method obtained very high precision and recall statistics. Compared to Kallisto, our method performs better in terms of precision and comparable in terms of TVD, while Kallisto performs better in terms of recall. Gene-by-gene boxplots for our method and Kallisto are available in Additional file 1.

**Table 1:** Average and standard deviation of precision, recall and TVD for each gene of the *Borellia* MLST scheme (B-MLST) and Kallisto, across all parameters combination

Precision	clpA	clpX	nifS	pepX
B-MLST	0.99 ±0.009	0.98 ±0.012	0.96 ±0.024	0.96 ±0.016
Kallisto	0.97 ±0.014	0.94 ±0.014	0.89 ±0.027	0.93 ±0.03
Recall				
B-MLST	0.95 ±0.022	0.94 ±0.027	0.90 ±0.05	0.94 ±0.034
Kallisto	0.99 ±0.004	0.99 ±0.005	0.99 ±0.003	0.99 ±0.006
TVD				
B-MLST	0.077 ±0.015	0.080 ±0.01	0.119 ±0.039	0.087 ±0.024
Kallisto	0.029 ±0.011	0.041 ±0.015	0.085 ±0.028	0.046 ±0.022
Precision	pyrG	recG	rplB	uvrA
B-MLST	0.97 ±0.024	0.98 ±0.013	0.99 ±0.007	0.98 ±0.011
Kallisto	0.93 ±0.02	0.89 ±0.021	0.95 ±0.012	0.93 ±0.023
Recall				
B-MLST	0.92 ±0.032	0.95 ±0.028	0.94 ±0.043	0.96 ±0.026
Kallisto	0.98 ±0.006	0.99 ±0.011	0.99 ±0.006	0.99 ±0.005
TVD				
B-MLST	0.110 ±0.019	0.082 ±0.028	0.089 ±0.03	0.069 ±0.02
Kallisto	0.0047 ±0.018	0.068 ±0.018	0.032 ±0.011	0.05 ±0.022

**SDP and full pipeline simulation.**The results are presented in Table 2. Given perfect input data, our SDP algorithm performed extremely well for each mechanism, maintaining a precision and recall of almost 75% with EvoMod3, the model that involves recombination. For the full pipeline simulation, our pipeline performs extremely well on the ADP, which is consistent with our observations in the ADP simulation. However, the full pipeline’s performance suffered in the SDP. Soft precision and recall are still high, but exact precision and recall are much lower. We can observe a dramatic impact on the SDP from relatively small errors in the ADP (i.e. wrong allele identification or discrepancy in the allele proportion estimation).

**Table 2:** Average and standard deviation of different statistics for each evolutionary mechanisms

	Soft-Precision	Soft-Recall	EMD	Precision	Recall	TVD
EM1	0.98 ±0.11	0.96 ±0.13	0.64 ±1.7	0.85 ±0.28	0.86 ±0.23	0.15 ±0.29
EM2	0.96 ±0.12	0.98 ±0.076	0.71 ±1.18	0.81 ±0.21	0.88 ±0.14	0.17 ±0.22
EM2e	0.98 ±0.11	0.97 ±0.1	0.34 ±0.81	0.91 ±0.20	0.92 ±0.17	0.1 ±0.23
EM2n	0.96 ±0.13	0.95 ±0.12	0.6 ±1.35	0.86 ±0.23	0.88 ±0.16	0.14 ±0.25
EM3	0.90 ±0.17	0.88 ±0.13	4.6 ±7.58	0.76 ±0.21	0.76 ±0.17	0.22 ±0.24
	ADP-Precision	ADP-Recall	ADP-TVD			
EM1	0.96 ±0.07	0.91 ±0.09	0.07 ±0.058			
EM2	0.93 ±0.07	0.91 ±0.07	0.26 ±0.16			
EM2e	0.93 ±0.08	0.91 ±0.08	0.34 ±0.25			
EM2n	0.92 ±0.09	0.9 ±0.09	0.34 ±0.25			
EM3	0.94 ±0.07	0.92 ±0.08	0.29 ±0.15			
	Soft-Precision	Soft-Recall	EMD	Precision	Recall	TVD
EM1	0.96 ±0.14	0.99 ±0.079	4.1 ±7.0	0.44 ±0.34	0.58 ±0.40	0.62 ±0.37
EM2	0.79 ±0.21	0.91 ±0.16	68.8 ±74.6	0.32 ±0.19	0.44 ±0.27	0.78 ±0.2
EM2e	0.72 ±0.24	0.88 ±0.22	98.9 ±89.4	0.36 ±0.26	0.5 ±0.30	0.72 ±0.26
EM2n	0.76 ±0.23	0.9 ±0.19	98.6 ±90	0.36 ±0.25	0.52 ±0.30	0.71 ±0.24
EM3	0.68 ±0.20	0.79 ±0.2	83.7 ±64	0.29 ±0.2	0.35 ±0.22	0.83 ±0.16

(Top) SDP simulation (Middle/Bottom) Full pipeline simulation: (Middle) ADP statistics, (Bottom) SDP statistics

**Comparison to strainEST.**We compared our methods to strainEST in the full pipeline simulation with 2 sets of experiments: (1) benchmark simulation where only existing strains are simulated (2) 4 different evolutionary mechanisms, where novel strains are involved. Our method outperforms strainEST in all situations. We refer the readers to the Additional file 1 for the detailed results.

### Application to real data

The sequencing data we analyzed are from 24 tick samples infected with *B. burgdorferi*, collected using the standard tick dragging method [24] in 2007 from 8 different sites in Vermont, New York, Massachusetts and Connecticut. For each tick sample, the *B. burgdorferi* genome was captured as described in [9]. The sequencing data is composed of 2×76bp paired-end reads and the number of read pairs ranges from 2.7·10^4^ to 2.7·10^6^ over all tick samples (coverages ranging from 5X to 500X).

**Fig. 2:**
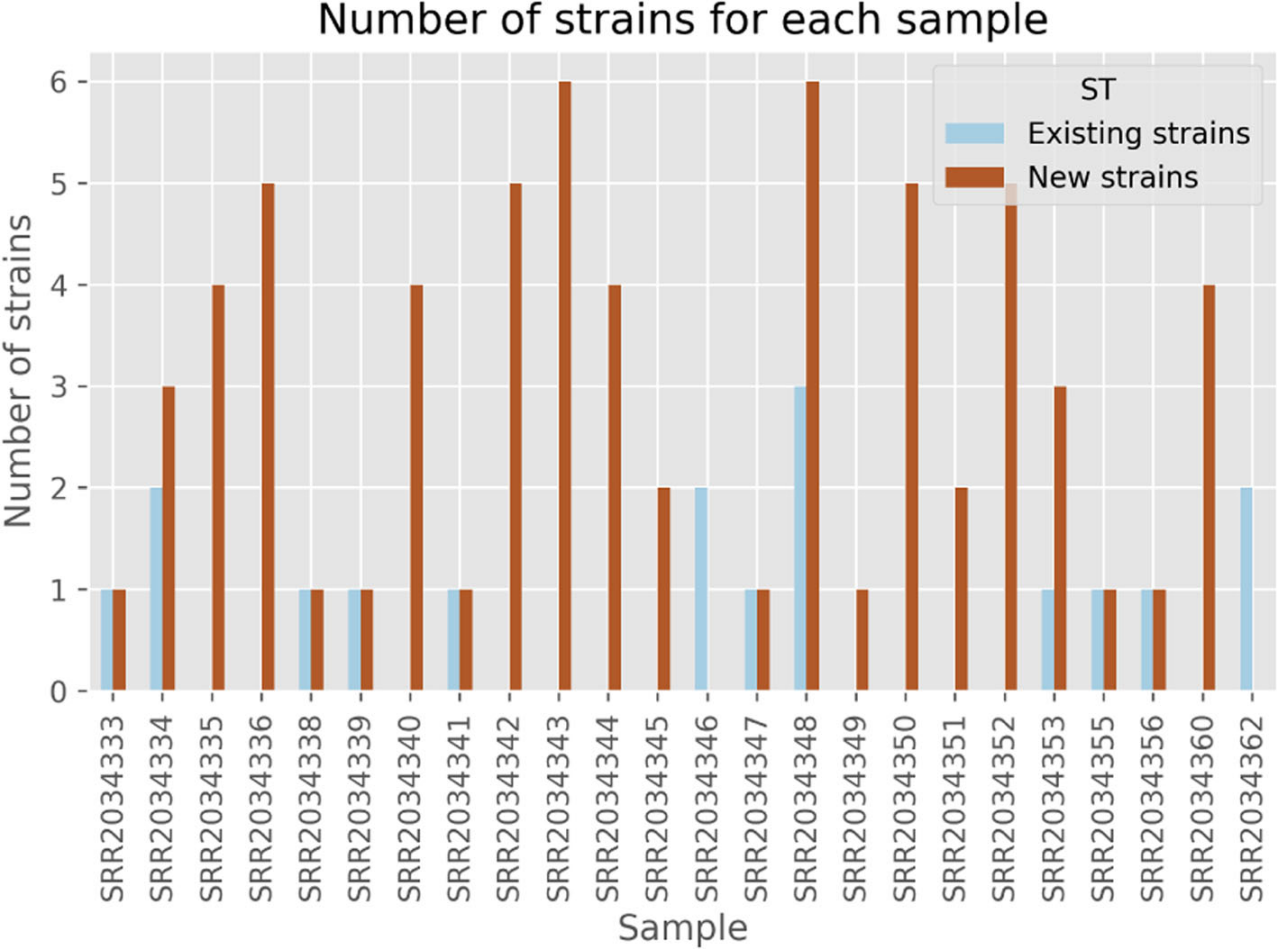
Distribution of the number of existing and novel strains per tick sample

**Fig. 3:**
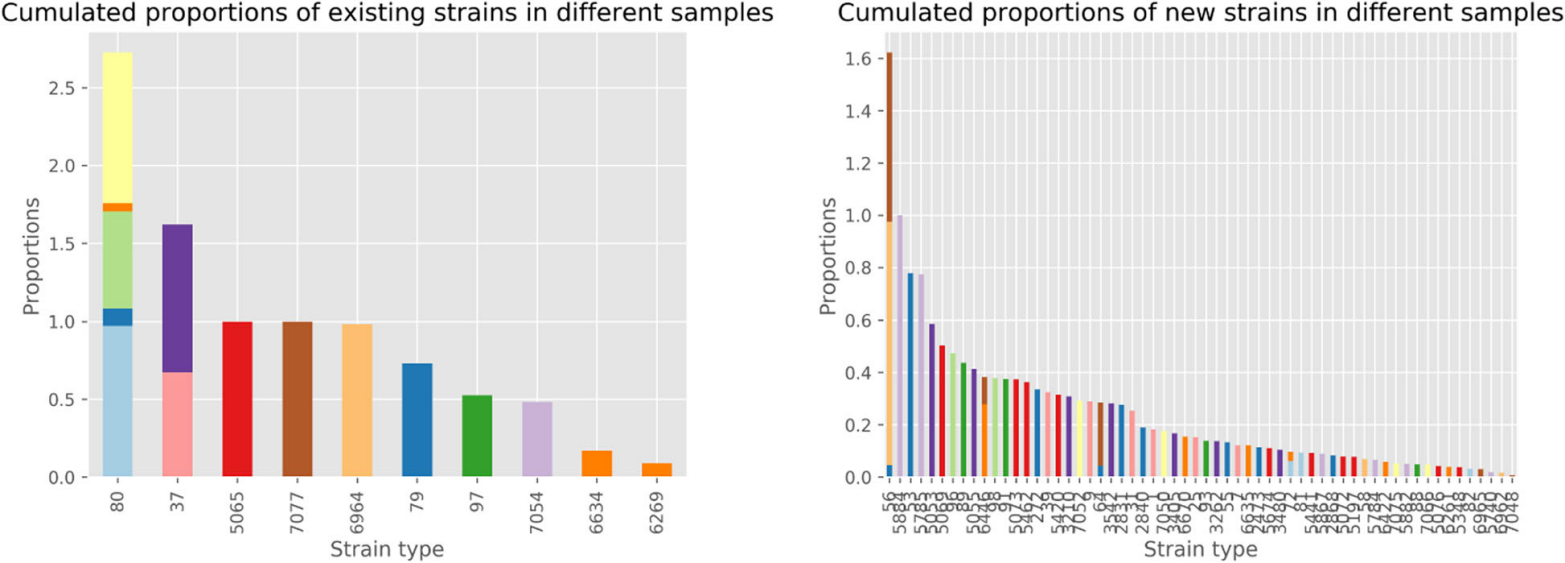
(Left) Cumulative proportion of the 10 existing strains in all 24 samples (within each bar, different colors represent different samples). (Right) Similar graph for the 60 novel strains

Based on the output of the pipeline, 60 novel and 10 existing strains were inferred to be potential candidates for explaining the strain diversity in this large sample of ticks. The total error component of the objective function of the MILP solving the SDP amounts to 1.258, or an average of 0.05 per sample. The total proportion of new strains is 14.67 in these 24 samples, for an average of 61%. For each sample having novel strains, 76% of its genotype is composed of novel strains. Figure 2 further illustrates the diversity, showing a wide range of strain composition in each of the 30 samples, with an average of 3 strains and a maximum of 9 strains infecting each sample, consistent with previous reports [5]. This suggest that the diversity of the *B. burgdorferi* strain types might be much larger than what was known so far. To further refine our analysis, Fig. 3 illustrates the distribution of strain types in the 30 tick samples and the respective contribution to the total diversity of each strain type. Although we observe that 2 of the 10 detected existing strains are present in more than one sample, only 5 out of the 60 novel strains appear in more than one sample.

It is striking to observe that most strain types appear in exactly one tick sample each. We can also observe that for 11 of the 24 samples, we do not detect any existing strains. This suggests that some of these strain types could have been improperly called, and that the correct call should have been another strain type, extremely close to this one in terms of sequence similarity; a reasonable cause for such errors could be a mistake while solving the ADP, in which case a wrongly called allele could be very similar to the correct allele. Due to possibility of wrong allele calls leading to introducing novel strains, we also computed a minimum spanning tree (MST) of the 70 strains found in these 24 samples, with edges weighted by the edit distance between the sequences of the alleles over the 8 genes of the MLST scheme. The MST figures are provided in Additional file 1. We can observe clusters of predicted strains that are very close to each other, such as, for example, a cluster of 8 novel strains and 2 existing strains that are all within edit distance 5 from each other. This suggests, in line with the level of precision and recall we observe in our simulations, that some of these strains might result from a limited level of erroneous allele calls, off by a couple of SNPs from the correct call, that result in this apparent high level of diversity.

## Conclusion

We presented an optimization-based pipeline for estimating the within-host strain diversity of a pathogen from WGS data analyzed in the MLST framework. This is a specific instance of estimating the diversity of a bacterial pathogen from metagenomics data, focusing on within-host diversity and taking advantage of the availability of a large database of known MLST strain types.

Our approach is composed of two main steps, each of a different nature; the first step detects the alleles present in a sample from the sequence data, while the second step estimates the strain diversity based on the output of the first one. In both steps we follow a parsimonious approach that aims at explaining the input using as few alleles or novel strains as possible. The main contribution of our work is the formulation and the solution of the Strain Diversity Problem for a group of samples. The main challenge of this problem is the need to consider a potentially large set of samples at once. While this leads to a relatively complex MILP, with a large number of variables (whose number is determined by the number of potentially present novel strain types), we believe that the ability to consider a large set of samples at once is an important part of the model, for example for analyzing sequencing data from pathogen hosts originating from a single geographical area. Our work shows that this problem, despite its complexity, can actually be solved to a good accuracy using reasonable amounts of computational resources.

Our experiments on real data suggest avenues for future research; in particular, the multiplicity of optimal solutions is obviously problematic, as calling a wrong allele in a single sample during the first step might force the MILP computing the strain types to introduce a new strain type. We can observe in our results on real data several groups of very closely related strain types, sometimes differing by a single SNP, which likely results from this issue. At the moment, our approach to this problem is to post-process the result of our pipeline to identify clusters of closely related strains, but other more principled approaches should be explored. Notwithstanding the aforementioned issues, our experiments suggest a strikingly high diversity in our dataset of 24 tick samples. This is not altogether surprising since the library of known strains might be limited, and within-host (or, more precisely, within-vector) evolution might result in the presence of a number of strains that only differ by a small number of SNPs in one or two loci of the MLST scheme.

Our work is, to our knowledge, the first comprehensive approach to the problem of reference-based detection of pathogen diversity in a collection of related samples that considers novel strain types. Our two-step pipeline, based on the principle of parsimony implemented through mixed integer linear programming, appears to perform extremely well on simulated data and produces reasonable results on a real dataset. We expect that both our approach and our publicly available pipeline will contribute to the development of accurate and efficient tools for quantifying the within-host diversity of bacterial pathogens.

## Supplementary information

**Additional file 1** Supplementary methods, figures and tables.

## Abbreviations

ADP: Allele Diversity problem
EMD: Earth-Mover’s Distance
FN: False Negative
FP: False Positive
ILP: Integer Linear Programming
MILP: Mixed Integer Linear Programming
MLST: Multi-Locus Sequence Typing
MST: Minimum Spanning Tree
NGS: Next-Generation Sequencing
SDP: Strain Diversity Problem
SNP: Single-Nucleotide Polymorphism
TN: True Negative
TP: True Positive
TVD: Total Variation Distance
WGS: Whole-Genome Sequencing

## Footnotes

1 https://www.ncbi.nlm.nih.gov/genome/genomes/738, accessed June 25, 2019.
