# Supplementary material for: Deconvoluting the diversity of within-host pathogen strains in a multi-locus sequence typing framework
Source: BMC Bioinformatics. 2019 Dec 17;20(Suppl 20):637. doi: 10.1186/s12859-019-3204-8 (PMC6915855; doi:10.1186/s12859-019-3204-8)
Supplement: Supplementary file 1 — Additional file 1 Supplementary methods, figures and tables. [file 12859_2019_3204_MOESM1_ESM.pdf]

Deconvoluting the Diversity of Within-host Pathogen Strains in a Multi-Locus Sequence Typing Framework
Supplementary Material

Guo Liang Gan [1], Elijah Willie [1], Cedric Chauve [2] and Leonid Chindelevitch [1]
1. School of Computing Science, Simon Fraser University, Burnaby, B.C. V5A 1S6, Canada.
2. Department of Mathematics, Simon Fraser University, Burnaby, B.C. V5A 1S6, Canada.

Complexity of the Allele Diversity Problem

Proof of NP-completeness of the Allele Diversity Problem

We transform the Allele Diversity Problem into a decision problem as follows.

Input:

- a set of reads $\mathcal{R} = \{r_1, r_2, \ldots\}$;
- a set of n alleles $\mathcal{A} = \{a_1, \ldots, a_n\}$ for the chosen locus;
- a target cost C.

Question: Is there a read cover whose cost is at most C?

It is clear that this problem is in NP, since given a particular subset $\mathcal{B}$ of alleles, it is easy to check, in polynomial time, that they form a read cover and that the cost of this read cover is at most C.

To show its NP-completeness we use a reduction from the 3-dimensional matching problem, formulated as follows:

Input:

- three disjoint sets, X, Y and Z, each of size n;
- a collection of $m \geq n$ triples $\{(x_i, y_i, z_i)\}_{1 \leq i \leq m}$, with $x_i \in X, y_i \in Y, z_i \in Z \; \forall \; i$.

Question: Is there a three-dimensional matching, i.e. a subset $M \subseteq \{1, 2, \ldots, m\}$ with $|M| = n$ such that $\cup_{i \in M}\{x_i, y_i, z_i\} = X \cup Y \cup Z$ (i.e. the corresponding triples contain each element exactly once)?

From a given input to the 3-dimensional matching problem we construct an instance of the allele diversity problem (with reads over a binary alphabet) as follows:

Select $3n$ binary strings, each of length $L-2$, such that the Hamming distance between any pair of them is at least 1 (for instance by using a Hamming code), pad them with a single 0 at the beginning and at the end to a total length L, and associate exactly one of them to each of the elements of $X \cup Y \cup Z$. For an element e denote by $s(e)$ the associated binary string.

Let the set of reads be $\mathcal{R} := \{s(e)\}_{e \in X \cup Y \cup Z}$. There are exactly $3n$ reads. We assume that the base quality scores at all the positions of all the reads equal the maximnum possible base quality score, so that after scaling, each mismatch contributes exactly 1 to the objective function.

For each triplet (x_i, y_i, z_i) we construct an allele of length $5L$, $s(x_i)1^L s(y_i)1^L s(z_i)$, where 1^L is the string of L 1's and the product is via concatenation. In other words, $\mathcal{A} := \{s(x_i)1^L s(y_i)1^L s(z_i)\}_{1 \leq i \leq m}$. Lastly, we set $C := n$. Since L can be chosen to be linear in n, and the codewords can be constructed in linear time with respect to n as well, this reduction can be performed in polynomial time.

We now prove the following

Claim: There is a read cover with cost at most n if and only if there is a three-dimensional matching.

Proof: By construction, $s(e)$ maps to any allele based on a triplet containing e with no errors, and to any allele based on a triplet not containing e with at least 1 error (since the encodings of the elements differ in at least one position, and a mapping overlapping with the "sentinel" string 1^L also incurs at least one mismatch due to the presence of a 0 at either end). It follows that we can get a score of at most n if we pick the n alleles corresponding to a three-dimensional matching, since there are no errors on any of the reads and n alleles are used in this case.

On the other hand, suppose that we do get a score of at most n. Since each allele can explain at most 3 reads with no errors, the case of no errors requires at least n alleles for the $3n$ reads, and the only way that this can be achieved with exactly n alleles is if each allele explains three different reads with no errors, meaning that the triples corresponding to the n chosen alleles form a three-dimensional matching. If there are $k > 0$ errors, then at most $n-k$ alleles can be used. It follows that at most $3(n-k)$ of the $3n$ reads incur no errors, and the remaining $3k$ reads must each incur at least one error, so the number of errors incurred must be at least $3k$, contradicting the hypothesis that there were only k errors. This proves the claim.

The ADP and the Uncapacitated Facility Location Problem

Our formulation of the ADP is very similar to that of the Uncapacitated Facility Location Problem (UFLP), in which a set of customers needs to be served by facilities placed in a subset of a finite set of possible locations, such that the total cost of opening the facilities plus the distances between each customer and the closest facility is minimized. Indeed, if we consider reads to be customers, alleles to be facilities, and distances to be the sum of normalized base quality scores of the corresponding mappings, the ADP becomes a special case of the UFLP. However, our problem is somewhat simpler than the general UFLP because the set of possible distances is limited. We exploit this fact in our ILP formulation which, unlike the formulation for the UFLP, has fewer variables and constraints.

Related to the Uncapacitated Facility Location Problem, already known to be NP-hard, our NP-hardness proof is of independent interest because it actually produces a set of reads and a set of alleles that have the desired distances, whereas not every instance of the UFLP may be obtainable from actual reads and alleles.

Alternative Formulation of the Allele Diversity Problem

The seemingly intuitive way to formulate the ADP is to introduce a decision variable b_{ij} representing the assignment of read r_i to allele a_j. Here we present the alternative formulation of ADP based on this idea as an integer linear program. Given there are n alleles and m reads, we denote the set of reads $R = \{r_1, ..., r_m\}$ and the set of alleles $A = \{a_1, ..., a_n\}$. Also, m_{ij} is the score for mapping read r_i to allele a_j, the entries of the matrix input for ADP as described previously. Next, the decision variables and objective function are as follows:

- $z_j = 1$ if allele a_j is chosen, and 0 otherwise.
- $b_{ij} = 1$ if the mapping of read r_i to allele a_j is chosen and 0 otherwise.
- min $\left(\sum_{i=1}^{m} \sum_{j=1}^{n} m_{ij} \cdot b_{ij} + \sum_{k=1}^{n} z_k \right)$

The constraints are:

- All reads are covered: $\sum_{j=1}^{n} b_{ij} = 1 \quad \forall i$
- If read r_i is assigned to allele a_j, then allele a_j must be chosen: $z_j \geq b_{ij} \quad \forall i, j$

Proof of the Equivalence of Both Formulations

Denote our formulation as (1) and the alternative formulation as (2). We prove that both formulations are equivalent by showing that a feasible solution of (1) can be translated to a feasible solution of (2), with both

having the same objective value and vice versa.
("$\Rightarrow$") Prove that a feasible solution of (1) can be translated into a feasible solution of (2). Given a feasible solution $s_1 = (\mathbf{x}; \mathbf{Y})$ where $\mathbf{x} = (x_j)$ and $\mathbf{Y} = (y_{ik})$. We denote a solution for (2), $s_2 = (\mathbf{z}; \mathbf{B})$ where $\mathbf{z} = (z_j)$ and $\mathbf{B} = (b_{ij})$. Also, recall that the matrix input for the ADP is $M = (m_{ij})_{m \times n}$.

- For all j, assuming the indices for both refer to the same allele, let $z_j = x_j$.
- For all i, due to the 2nd constraint in (1), $\exists!\ k^*$ such that $y_{ik^*} = 1$ and $y_{ik} = 0\ \forall k \neq k^*$. Denote $H_{ik^*} = \{j \mid m_{ij} = q_{ik^*} \wedge x_j = 1, j \in \{1, ..., n\}\}$. Choose an index $j^* \in H_{ik^*}$, let $b_{ij^*} = 1$ and $b_{ij} = 0$ for all $j \in H_{ik^*} - \{j^*\}$. For $j \notin H_{ik^*}$, let b_{ij}=0.

Claim: s_2 is a feasible solution for (2) with same objective value as (1).
Proof:

- Constraint 1: $\sum_{j=1}^{n} b_{ij} = 1\ \forall i$. This is true by construction of b_{ij}.
- Constraint 2: $z_j \geq b_{ij}\ \forall i, j$. If $b_{ij} = 1$, then $j = j^* \Rightarrow j \in H_{ik^*} \Rightarrow x_j = 1 = z_j \Rightarrow z_j \geq b_{ij}$. The case for $b_{ij} = 0$ is trivial as z_j is always ≥ 0.
- Objective value: The summation of the allele variables is the same in both formulations. For the other summation term:

$$\sum_{i=1}^{|\mathcal{R}|} \sum_{k=1}^{|M_i|} q_{ik} y_{ik} = \sum_{i=1}^{|\mathcal{R}|} \left(q_{ik^*} y_{ik^*} + \sum_{k \neq k^*} q_{ik} y_{ik} \right)$$
$$= \sum_{i=1}^{|\mathcal{R}|} \left(m_{ij^*} b_{ij^*} + \sum_{j \in \{1,..,n\} - \{j^*\}} m_{ij} b_{ij} \right) \qquad (b_{ij} = 0\ , j \neq j^*)$$
$$= \sum_{i=1}^{m} \sum_{j=1}^{n} m_{ij} b_{ij}$$

("$\Leftarrow$") Prove that a feasible solution of (2) can be translated to a feasible solution of (1). Given a feasible solution $s_2 = (\mathbf{z}; \mathbf{B})$.

- Let $x_j = z_j$
- For all i, due to first constraint of (2), $\exists! j \in \{1, ..., n\}$, say j^*, where $b_{ij^*} = 1$. Also, say the corresponding score $q_{ik^*} = m_{ij^*}$. Let $y_{ik^*} = 1$, $y_{ik} = 0\ \forall q_{ik} \in M_i - \{q_{ik^*}\}$.

Claim: s_2 is a feasible solution for (2) with same objective value as (1).
Proof:

- Constraint 1: If $y_{ik} = 1$, $k = k^*$. Due to how we construct the solution, $\exists j^*$ such that $b_{ij^*} = 1 \wedge m_{ij^*} = q_{ik^*}$. Due to the 2nd constraint of (2), $x_j^* = z_j^* \geq b_{ij^*} = 1 = y_{ik^*}$. The case of $y_{ik} = 0$ is trivial.
- Constraint 2: $\sum_{k=1}^{|M_i|} y_{ik} = \left(y_{ik^*} + \sum_{k \neq k^*} y_{ik} \right) = (1 + 0) = 1$
- Objective value: The summation of the allele variables is the same. For the other summation term:

$$\sum_{i=1}^{m} \sum_{j=1}^{n} w_{ij} b_{ij} = \sum_{i=1}^{m} \left(b_{ij^*} w_{ij^*} + \sum_{j \neq j^*} w_{ij} b_{ij} \right)$$
$$= \sum_{i=1}^{m} \left(q_{ik^*} y_{ik^*} + \sum_{k \neq k^*} q_{ik} y_{ik} \right) \qquad (\text{as } y_{ik} = 0 \text{ for } k \neq k^*)$$
$$= \sum_{i=1}^{|\mathcal{R}|} \sum_{k=1}^{|M_i|} q_{ik} y_{ik}$$

Proof of NP-completeness of the Strain Diversity Problem

We turn the Strain Diversity Problem into a decision problem by putting the objective function value into the input. This decision problem version is then formulated as follows:

Input:

- The set $G_{ij} = \{g_{ij1}, g_{ij2}, \ldots\}$ of all alleles selected for locus j in sample i, together with the set $P_{ij} = \{p_{ij1}, p_{ij2}, \ldots\}$ of proportions of these alleles;
- A database Ω of known strain types;
- An error bound $\epsilon \in [0, 1]$;
- A target cost C.

Let the set of all possible strain types for sample i, the Cartesian product $G_{i1} \times G_{i2} \times \ldots \times G_{i\ell}$, be denoted by $V_i = \{V_{i1}, V_{i2}, \ldots, V_{iH_i}\}$ with $H_i = \prod_{j=1}^{\ell} |G_{ij}|$. For simplicity, assuming the weights for each novel strain are set to 1.

Question: Is there a set of proportions π_{ih} for each strain type $V_{ih} \in V_i$ in each sample i, satisfying the validity constraint

$$\nu_{ijk} := \sum_{(i,k)|g_{ijk} \in V_{ik}} \pi_{ik} \in \left[p_{ijk} - \epsilon, p_{ijk} + \epsilon\right] \; \forall \; g_{ijk} \in G_{ij},$$

such that the total cost is at most equal to the target C, i.e.

$$|\{V_{ih}|\pi_{ih} > 0\} - \Omega| + \sum_{i,j,k} |p_{ijk} - \nu_{ijk})| \leq C?$$

This problem is clearly in NP because given a set of proportions π_{ih}, it is easy to check, in linear time in the size of the input, that the validity constraints are satisfied, and that the cost is at most C.

To show its NP-completeness we use a reduction from the 3-partition problem, formulated as follows:

Input: A multiset of $n = 3m$ positive integers $x_1, x_2, \ldots, x_n$ with sum $S = mB$ for some integer B, and satisfying $\frac{B}{4} < x_i < \frac{B}{2}$ for each $1 \leq i \leq n$.

Question: Does there exist a partition of this multiset into m disjoint triples $T_1, T_2, \ldots, T_m$, such that the sum of each triple is exactly B?

From a given input to the 3-partition problem we construct the following instance of SDP. Let us choose $\epsilon = \frac{1}{4m}$. Let us look at a single sample with two loci, 1 and 2, where the first locus has the $n = 3m$ possible alleles $g_1, g_2, \ldots, g_n$ and the second one has the m possible alleles $h_1, h_2, \ldots, h_m$.

Let the target allele distribution for locus 1 be g_1 with proportion $\frac{x_1}{S}$, g_2 with proportion $\frac{x_2}{S}, \ldots, g_n$ with proportion $\frac{x_n}{S}$, and let the target allele distribution for locus 2 be h_i with proportion $\frac{1}{m}$ for each $1 \leq i \leq m$.

Finally, let the library strains be $\Omega := \emptyset$, and let the target cost be $C := n$.

It is clear that the transformation is polynomial in the input size. We now prove the following

Claim: The original problem has a "yes" answer if and only if the transformed version has cost at most C.

Proof: Suppose that there is a partition of the multiset into disjoint triples $T_1, T_2, \ldots, T_m$. Then we can take the 3 integers, say $x_{j_1}, x_{j_2}, x_{j_3}$, in a triple T_j, and use the 3 new strains $g_{j_1} - h_j$ with proportion $\frac{x_{j_1}}{S}$, $g_{j_2} - h_j$ with proportion $\frac{x_{j_2}}{S}$, and $g_{j_3} - h_j$ with proportion $\frac{x_{j_3}}{S}$, to cover the allele h_j completely, with no error (since $\frac{x_{j_1}}{S} + \frac{x_{j_2}}{S} + \frac{x_{j_3}}{S} = \frac{B}{S} = \frac{1}{m}$ by construction). Thus, we need a total of $n = 3m$ strains to cover all the alleles in both loci with no error, so the cost of our solution is indeed n.

Conversely, suppose that there is a solution with cost at most n. Note first that because $\epsilon = \frac{1}{4m} = \frac{B}{4Bm} = \frac{B}{4S} < \frac{x_i}{S}$ for any $1 \leq i \leq n$, it follows that no allele in locus 1 can be eliminated from the solution while satisfying the validity constraint. It follows that at least $n = 3m$ strains are needed in any valid solution, so the cost cannot be smaller than n.

Furthermore, in order to get a cost of exactly n, there needs to be no error on the proportions and exactly $n = 3m$ strains, so each allele in locus 1 must be in exactly one strain. Thus, the allele h_j for locus 2 must be associated with a subset T_j of alleles of locus 1, and the T_j must be disjoint and therefore form a partition of the alleles of locus 1. Since $\frac{B}{4} < x_i < \frac{B}{2}$ for each i, we must have exactly 3 alleles in each subset T_j, since

adding up 2 of the $\frac{x_i}{S}$ results in a sum below the target proportion $\frac{B}{S} = \frac{1}{m}$ for h_j, and adding up 4 of them results in a sum above it, thus incurring a non-zero error on the proportions.

By considering the sum of the proportions in each triple T_j containing three alleles, say $g_{j_1}, g_{j_2}, g_{j_3}$, we see that we must have

$$\frac{x_{j_1}}{S} + \frac{x_{j_2}}{S} + \frac{x_{j_3}}{S} = \frac{1}{m} = \frac{B}{S} \Rightarrow x_{j_1} + x_{j_2} + x_{j_3} = B.$$

As the T_j are disjoint, this proves that the answer to the original problem is "yes", completing the proof.

WGS data preprocessing.

Before running the pipeline, we map the reads of each sample to a database of alleles for each gene. We use the memory efficient short read mapper Bowtie (v0.12.7) for read mapping. Next, we use *SAMTOOLS* (v1.3.1) to process the *SAM* file and extract information such as reads that are mapped as a pair, the alleles that are involved in the mappings, the number of mismatches, the base qualities, and the position of the mismatches.

Definition of Earth-Mover's Distance

Let $\mathcal{T} = \{T_i, t_i\}_{i=1}^{|\mathcal{T}|}$ be the set of true strains T_i with its respective proportion t_i and $\mathcal{P} = \{P_j, p_j\}_{i=1}^{|\mathcal{P}|}$ be the set of predicted strains P_j with predicted proportion p_j. Also, denote d_{ij} as the edit distance between T_i and P_j. EMD measures the minimum amount of work to transform the predicted distribution defined by $\mathcal{P}$ into true distribution defined by $\mathcal{T}$, where the amount of work is also weighted by distances d_{ij}. It involves solving a transportation problem which can be modeled as a network flow problem, where the problem is to obtain a flow $F = \{f_{ij}\}$ which minimizes $\sum_{ij} f_{ij} d_{ij}$ and the $EMD = \frac{\sum_{ij} f_{ij} d_{ij}}{\sum_{ij} f_{ij}}$. It can be thought as a network flow problem in the following sense: we assign fractions of each P_i to any T_j (flow f_{ij}), constrained by the maximum amount of fractions that P_i and T_j can give and take, which is p_i and t_j (capacities), where the assignments f_{ij} are weighted by d_{ij}.

Results of Kallisto on ADP simulation

As Kallisto introduces large number of alleles with extremely small abundances, we only retained alleles with larger than 1% proportion.

**Prec.**	clpA	clpX	nifS	pepX	pyrG	recG	rplB	uvrA
AVG	0.97	0.94	0.89	0.93	0.93	0.89	0.95	0.93
SD	0.014	0.014	0.027	0.03	0.02	0.021	0.012	0.023
**Recall**								
AVG	0.99	0.99	0.99	0.99	0.98	0.99	0.99	0.99
SD	0.004	0.005	0.003	0.006	0.006	0.011	0.006	0.005
**TVD**								
AVG	0.029	0.041	0.085	0.046	0.0047	0.068	0.032	0.05
SD	0.011	0.015	0.028	0.022	0.018	0.018	0.011	0.022

Table 1: Kallisto: Average and standard deviation of precision, recall and TVD for each gene of the *Borellia* MLST scheme across all parameters combination.

Results of strainEST on full pipeline simulation

Recall that full pipeline simulation involves generating reads after strains are chosen, and our pipeline first solves the ADP problem then the SDP problem, by taking results of ADP as an input. StrainEST infers strains and abundances, and we compute the statistics for ADP based on the strains and abundances inferred. In this section, we present the results for our algorithm and strainEST on 2 different set of experiments: (1) benchmark simulation: Only existing strains are chosen for simulation, where we consider $k = \{1, 2\}$ number of strains (2) EvoMods simulation: 4 evolutionary mechanisms. **Remark**: As strainEST can only take a maximum of 100 genomes, we randomly choose 100 existing strains as the database for simulation.

Results on benchmark simulation

	Our Algorithm			StrainEST		
	Precision	Recall	TVD	Precision	Recall	TVD
k=1	0.96 ± 0.057	0.88 ± 0.08	0.052 ± 0.052	0.67 ± 0.29	0.5 ± 0.47	0.4 ± 0.33
k=2	0.94 ± 0.061	0.9 ± 0.06	0.33 ± 0.23	0.62 ± 0.24	0.5 ± 0.23	0.4 ± 0.274

Table 2: Comparison between our algorithm and strainEST on the SDP problem

	SoftPrecision	SoftRecall	EMD	Precision	Recall	TVD
k=1, our method	0.99 ± 0.08	1 ± 0	0.623 ± 0.625	0.48 ± 0.39	0.68 ± 0.47	0.406 ± 0.42
k=1, strainEST	0.35 ± 0.43	0.48 ± 0.5	8.07 ± 7.39	0.24 ± 0.4	0.3 ± 0.46	0.4 ± 0.33
k=2, our method	0.90 ± 0.18	0.99 ± 0.08	79.2 ± 90.3	0.4 ± 0.25	0.6 ± 0.31	0.67 ± 0.24
k=2, strainEST	0.37 ± 0.32	0.49 ± 0.34	12.5 ± 8.7	0.2 ± 0.24	0.28 ± 0.32	0.714 ± 0.352

Table 3: Comparison between our algorithm and strainEST on the ADP problem

Results on 4 EvoMods

The results for different evolutionary mechanisms presented for our algorithm are different from those presented in the Results section, due to the database size constraint by strainEST.

	StrainEST			Our Algorithm		
	Precision	Recall	TVD	Precision	Recall	TVD
EM1	0.55 ± 0.26	0.51 ± 0.34	0.5 ± 0.29	0.97 ± 0.06	0.92 ± 0.08	0.066 ± 0.059
EM2	0.64 ± 0.16	0.57 ± 0.24	0.42 ± 0.23	0.93 ± 0.07	0.91 ± 0.07	0.252 ± 0.16
EM2e	0.52 ± 0.16	0.46 ± 0.24	0.51 ± 0.22	0.94 ± 0.06	0.92 ± 0.06	0.277 ± 0.162
EM2n	0.61 ± 0.18	0.56 ± 0.23	0.41 ± 0.22	0.93 ± 0.07	0.92 ± 0.07	0.275 ± 0.168

	Soft-Precision	Soft-Recall	EMD	Precision	Recall	TVD
EM1	0.42 ± 0.46	0.28 ± 0.27	13.6 ± 10.9	0.27 ± 0.41	0.18 ± 0.24	0.83 ± 0.26
EM2	0.45 ± 0.31	0.33 ± 0.18	13.6 ± 7.84	0.3 ± 0.31	0.21 ± 0.18	0.79 ± 0.26
EM2e	0.22 ± 0.26	0.18 ± 0.17	17.86 ± 9.04	0.13 ± 0.23	0.1 ± 0.15	0.912 ± 0.178
EM2n	0.43 ± 0.32	0.38 ± 0.23	12.7 ± 7.68	0.26 ± 0.3	0.22 ± 0.22	0.8 ± 0.251

	Soft-Precision	Soft-Recall	EMD	Precision	Recall	TVD
EM1	0.87 ± 0.29	0.91 ± 0.27	3.5 ± 3.28	0.41 ± 0.34	0.54 ± 0.39	0.643 ± 0.37
EM2	0.74 ± 0.23	0.88 ± 0.21	55.58 ± 62	0.28 ± 0.23	0.39 ± 0.3	0.813 ± 0.195
EM2e	0.69 ± 0.27	0.83 ± 0.28	61.23 ± 65.32	0.28 ± 0.21	0.38 ± 0.28	0.766 ± 0.218
EM2n	0.79 ± 0.21	0.94 ± 0.15	60.6 ± 67	0.33 ± 0.24	0.47 ± 0.31	0.742 ± 0.243

Table 4: Average and standard deviation of different statistics for full pipeline simulation of 4 evolutionary mechanisms. (Top) ADP comparison (Middle) strainEST on SDP (Bottom) Our method on SDP

Accession numbers for the real data samples

The following table lists the SRA accession numbers of all the samples used in this study.

SRR2034333	SRR2034334	SRR2034335	SRR2034336	SRR2034338
SRR2034339	SRR2034340	SRR2034341	SRR2034342	SRR2034343
SRR2034344	SRR2034345	SRR2034346	SRR2034347	SRR2034348
SRR2034349	SRR2034350	SRR2034351	SRR2034352	SRR2034353
SRR2034355	SRR2034356	SRR2034360	SRR2034362	

Table 5: SRA accession numbers for all samples used in this study

Minimum Spanning Tree of Strains Found in 24 Samples

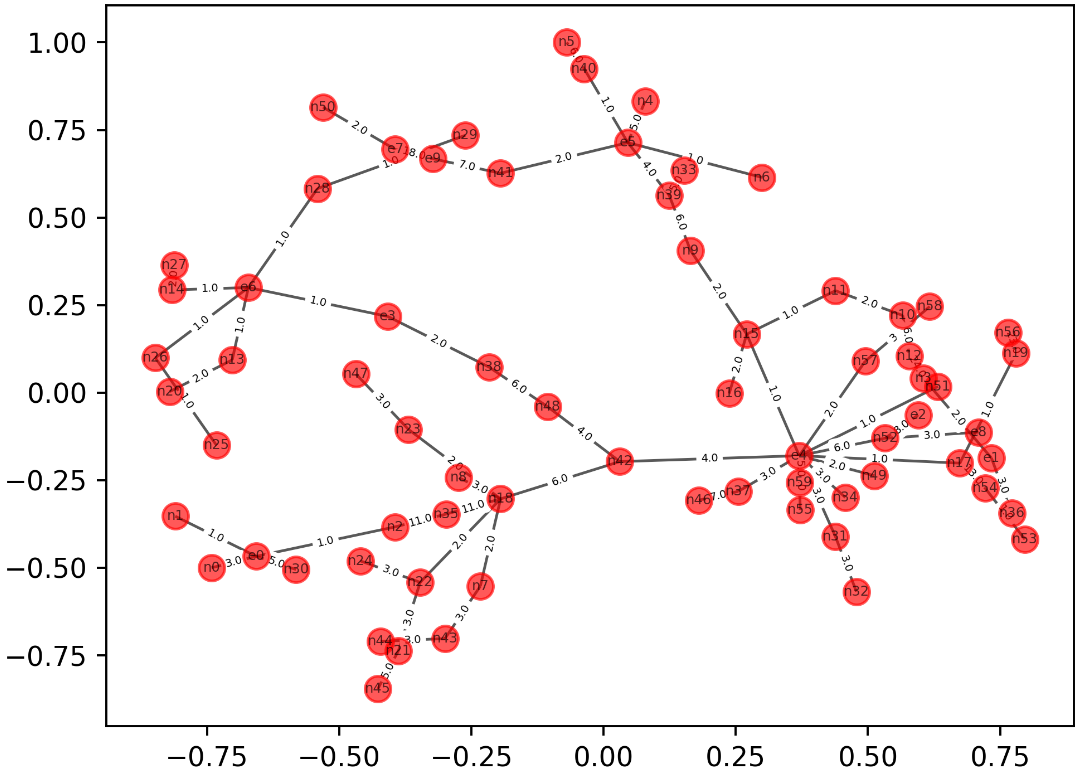

Figure 1: Nodes with prefix ’n’ represents novel strains, ’e’ represents existing.

We can observe clusters of strains which are very close to each other, for example, the circled tree branch in the upper left, consists nodes n25, n26, e6, e3, n13, n20, n14, n27, n28, n29. These nodes can be grouped as a cluster with strains of edit distance at most 5 bases. It is worth taking note that this distance is taken across 8 genes where each is approximately 580 bases long. Furthermore, mapping back to samples which contain these strains, n13 and n14 are in sample SRR2034335; n25, n26, n27, n28, n29 are in sample SRR2034342. Similar observations were seen on nodes which are in the star structure circled in Figure2 where any 2 nodes in the structure differ by at most 11 bases. These observation indicates that either strains in these samples mutate into extremely similar strains, or it indicates that wrong allele call leads to relatively higher number of novel strains inferred by our method.

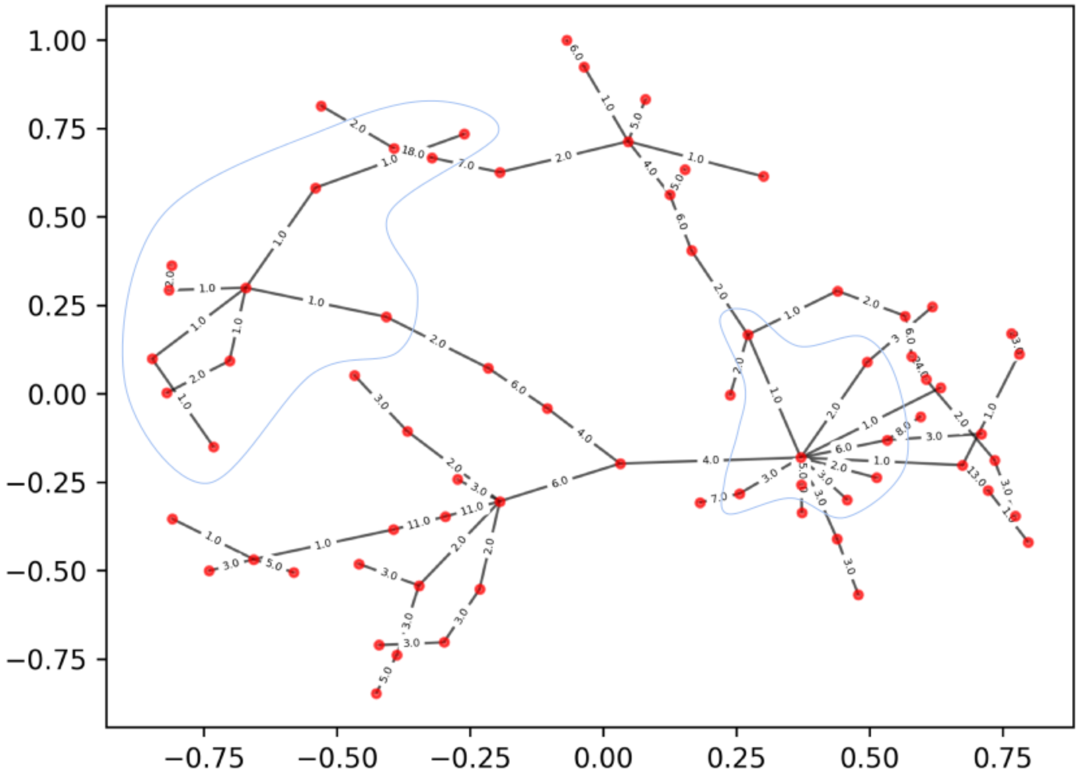

Figure 2: Minimum spanning tree of strains found in 24 samples.

Box Plots of Our Method on ADP simulation

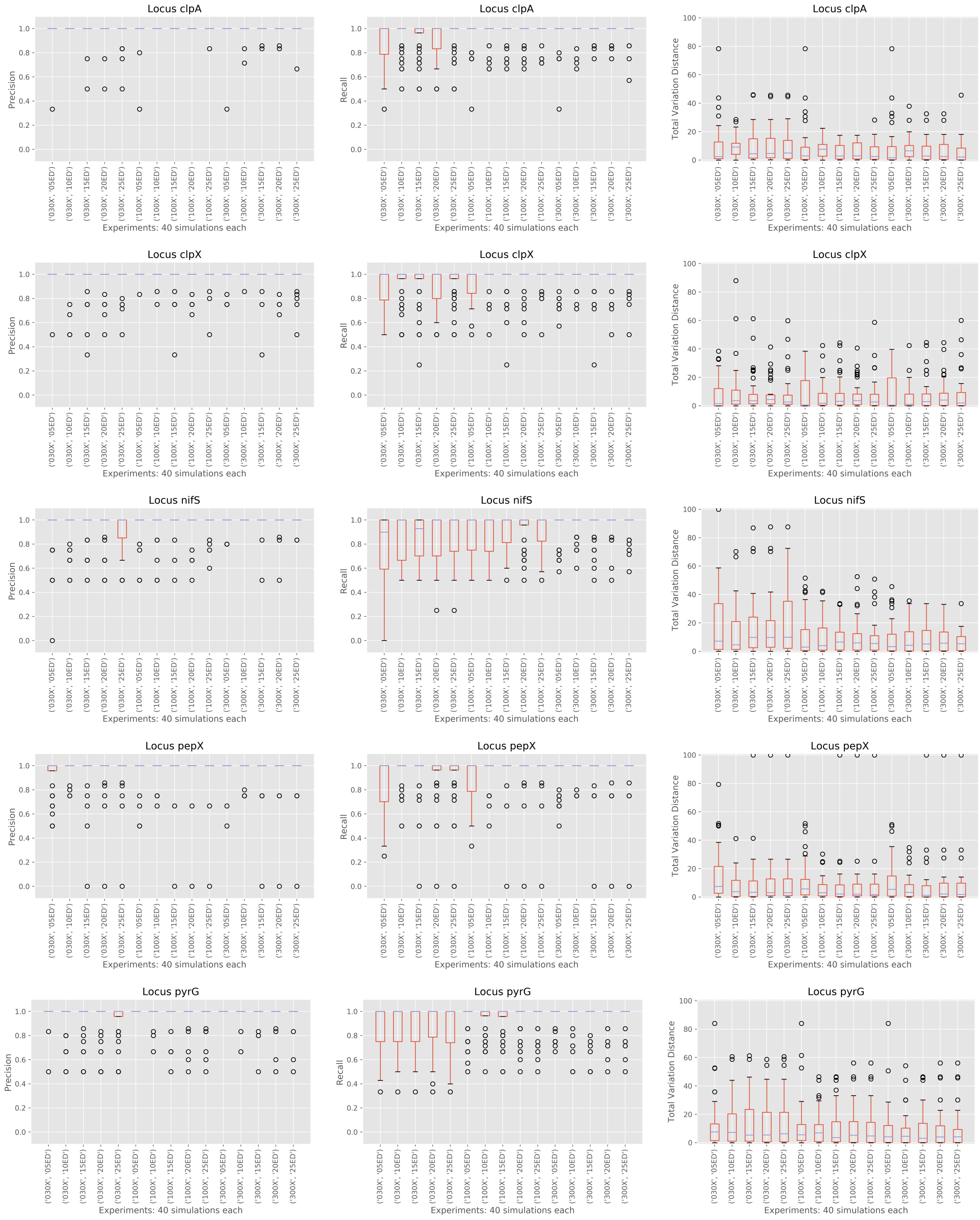

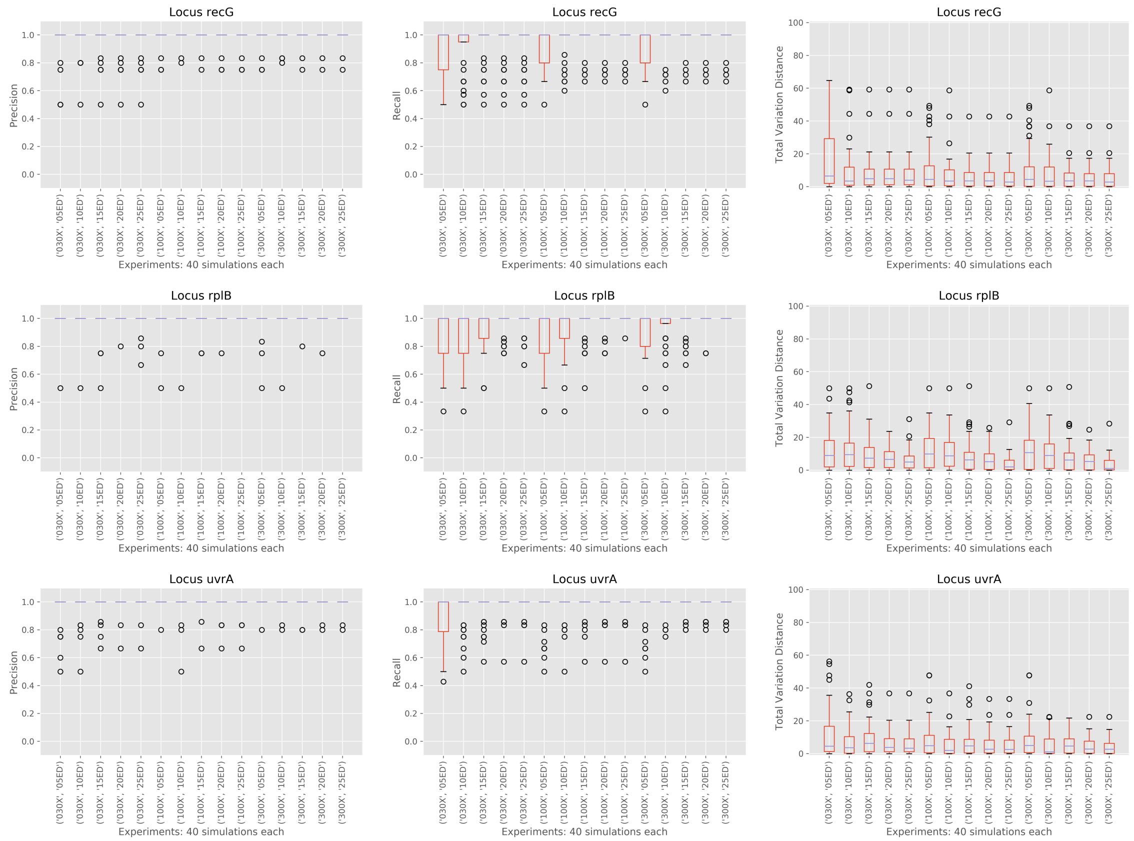

Figure 3: Box plots of Precision, Recall, and TVD of our method for different combinations of coverages and edit distance parameters over 40 simulations for 8 genes of the Borrelia MLST scheme

Box plots of Kallisto on ADP simulation

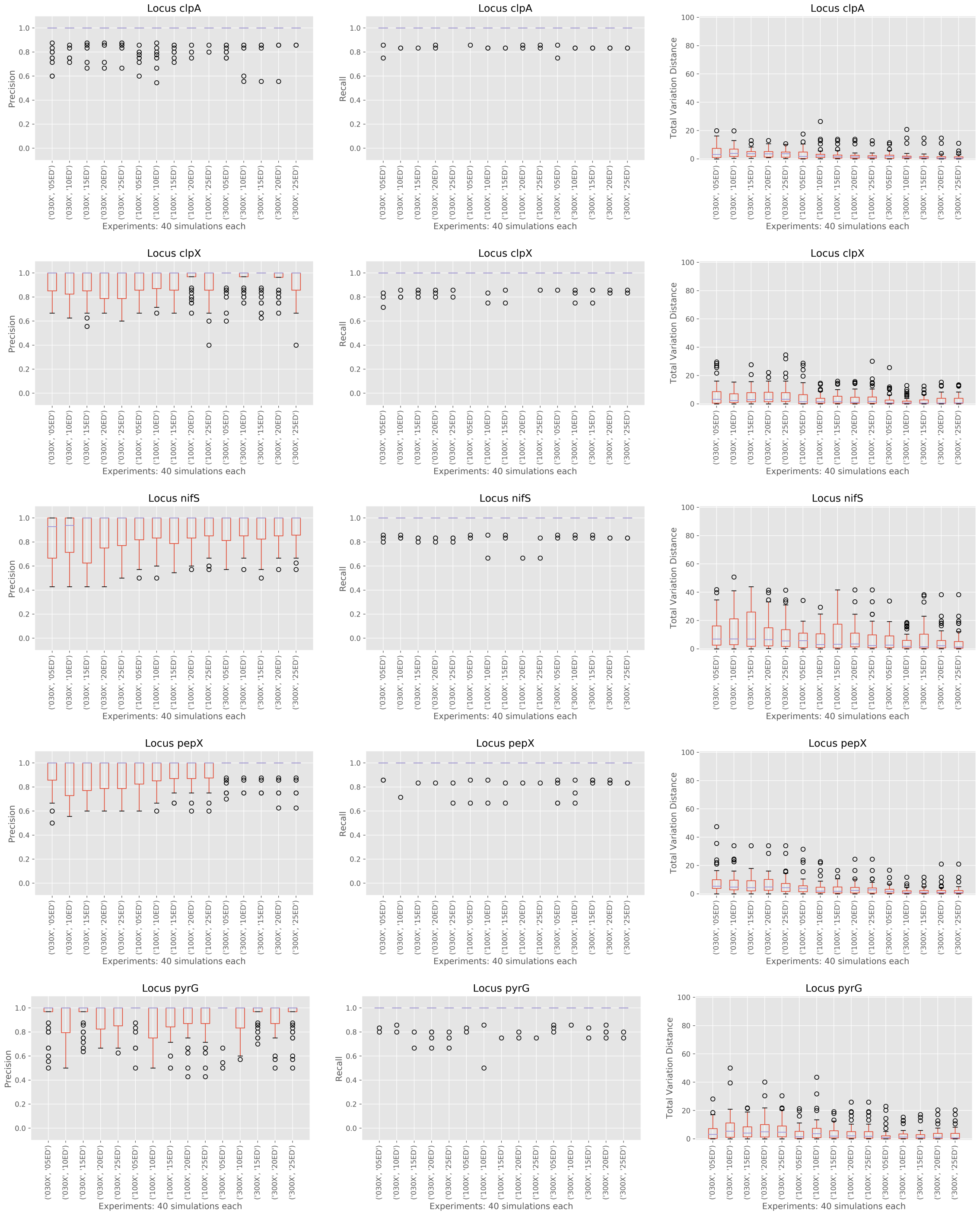

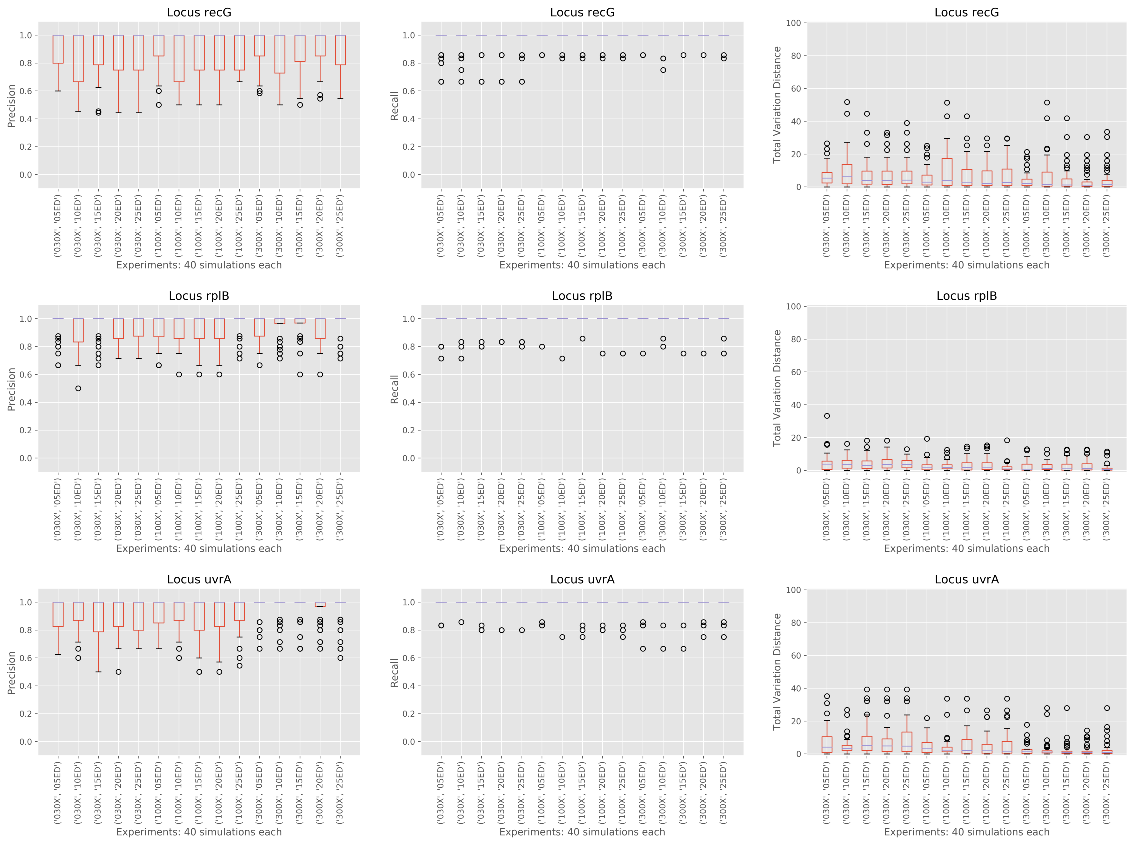

Figure 4: Box plots of Precision, Recall, and TVD of Kallisto for different combinations of coverages and edit distance parameters over 40 simulations for 8 genes of the Borrelia MLST scheme

Box Plots of Our Method and strainEST on Benchmark Simulation

Recall that the benchmark simulation is one of the simulations used to compare to strainEST.

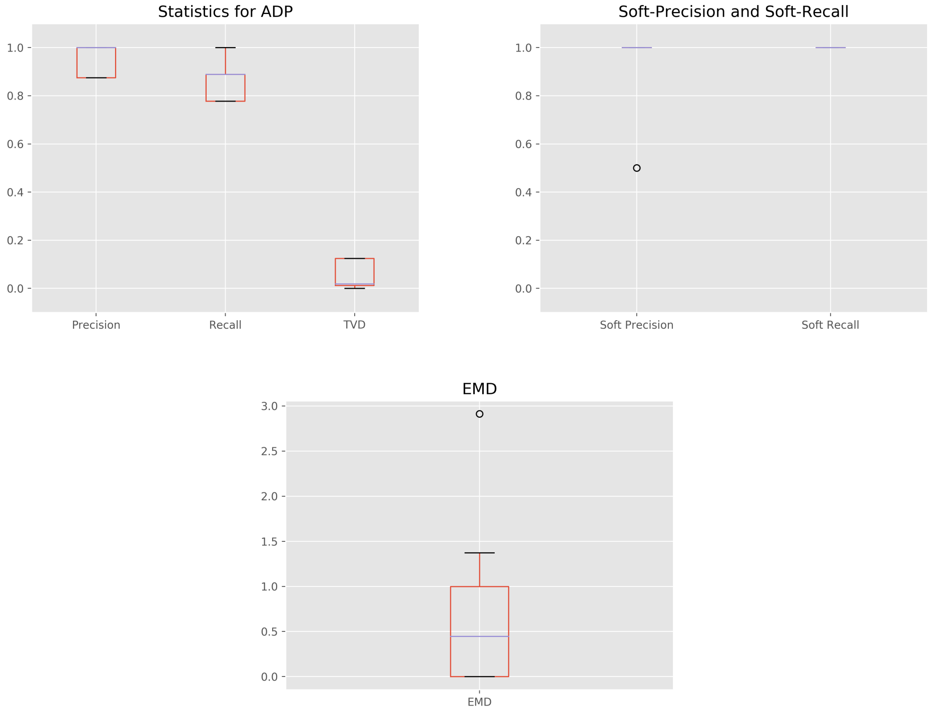

Figure 5: Our method: Box plots of (1) ADP statistics (2) Soft-Precision and Soft-Recall on SDP (3) EMD on SDP where k=1

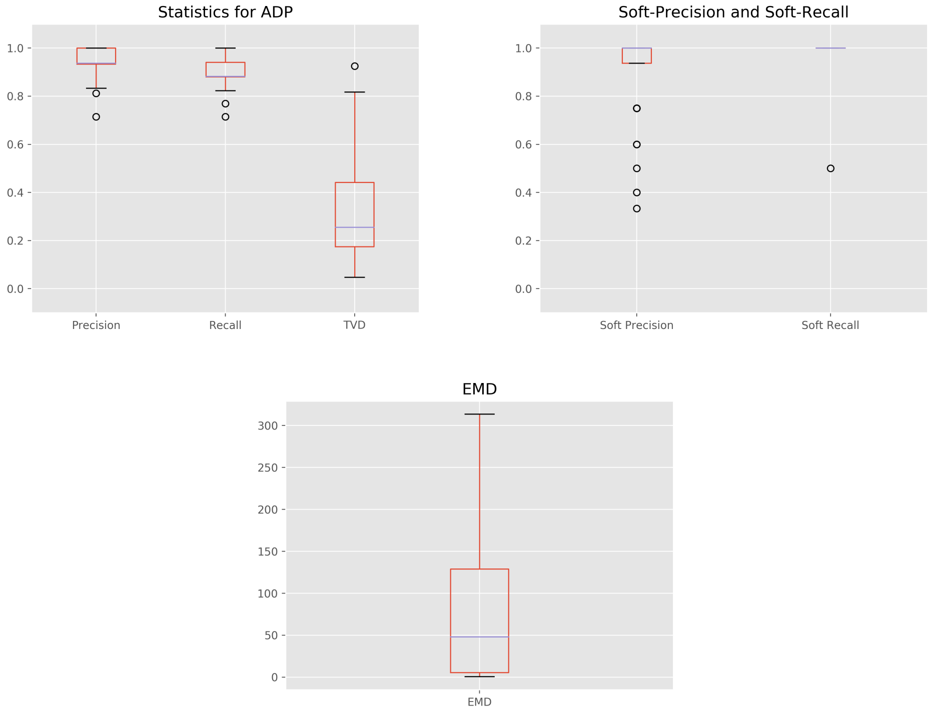

Figure 6: Our method: Box plots of (1) ADP statistics (2) Soft-Precision and Soft-Recall on SDP (3) EMD on SDP where k=2

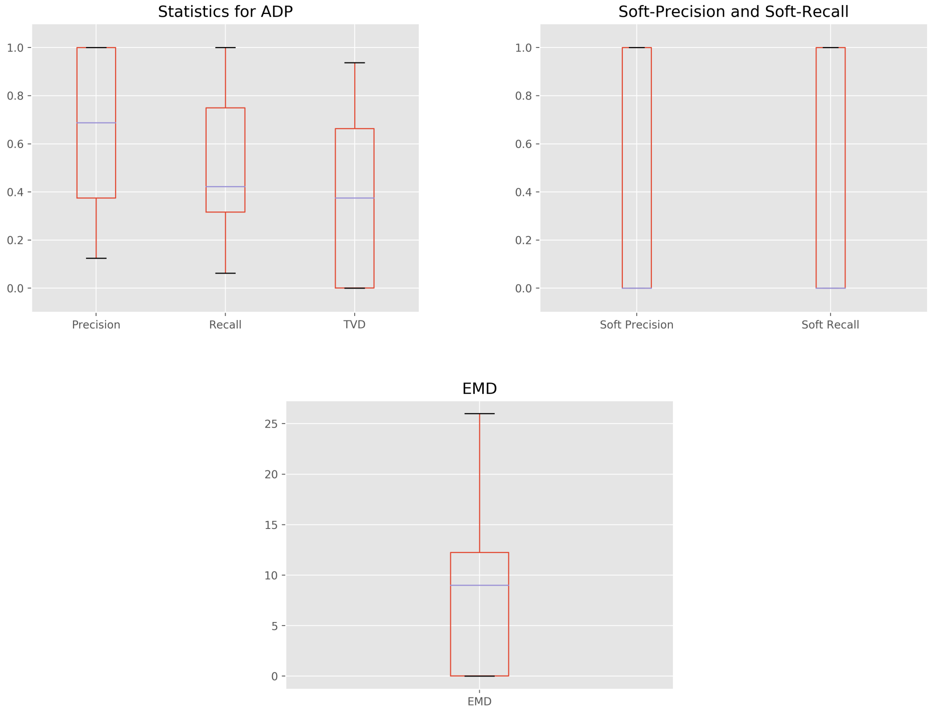

Figure 7: strainEST: Box plots of (1) ADP statistics (2) Soft-Precision and Soft-Recall on SDP (3) EMD on SDP where k=1

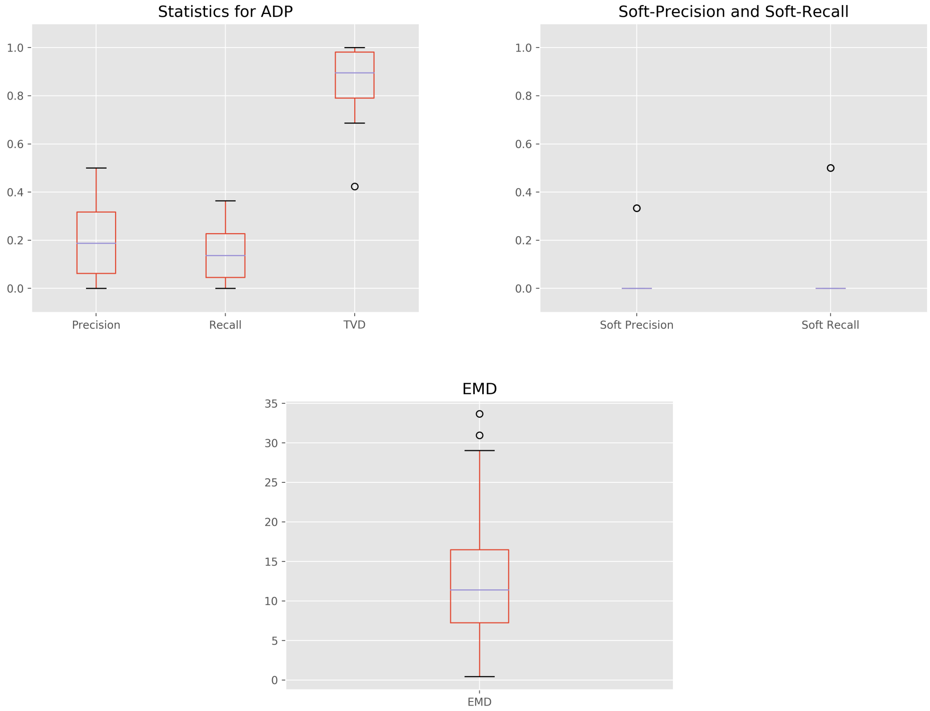

Figure 8: strainEST: Box plots of (1) ADP statistics (2) Soft-Precision and Soft-Recall on SDP (3) EMD on SDP where k=2
